# The Effect of the Feeding System on Fat Deposition in Yak Subcutaneous Fat

**DOI:** 10.3390/ijms24087381

**Published:** 2023-04-17

**Authors:** Lin Xiong, Jie Pei, Pengjia Bao, Xingdong Wang, Shaoke Guo, Mengli Cao, Yandong Kang, Ping Yan, Xian Guo

**Affiliations:** 1 Animal Science Department, Lanzhou Institute of Husbandry and Pharmaceutical Sciences, Chinese Academy of Agricultural Sciences, Lanzhou 730050, China; 2 Key Laboratory of Animal Genetics and Breeding on Tibetan Plateau, Ministry of Agriculture and Rural Affairs, Lanzhou 730050, China; 3 Key Laboratory for Yak Genetics, Breeding, and Reproduction Engineering of Gansu Province, Lanzhou 730050, China

**Keywords:** yak, feeding system, subcutaneous fat, fat deposition, regulatory mechanism

## Abstract

Fat deposition is very important to the growth and reproduction of yaks. In this study, the effect of the feeding system on fat deposition in yaks was explored by transcriptomics and lipidomics. The thickness of the subcutaneous fat in yaks under stall (SF) and graze feeding (GF) was evaluated. The transcriptomes and lipidomes of the subcutaneous fat in yaks under different feeding systems were detected by RNA-sequencing (RNA-Seq) and non-targeted lipidomics based on ultrahigh-phase liquid chromatography tandem mass spectrometry (UHPLC-MS), respectively. The differences in lipid metabolism were explored, and the function of differentially expressed genes (DEGs) was evaluated by gene ontology (GO) and Kyoto encyclopedia of genes and genome (KEGG) analysis. Compared with GF yaks, SF yaks possessed stronger fat deposition capacity. The abundance of 12 triglycerides (TGs), 3 phosphatidylethanolamines (PEs), 3 diglycerides (DGs), 2 sphingomyelins (SMs) and 1 phosphatidylcholine (PC) in the subcutaneous fat of SF and GF yaks was significantly different. Under the mediation of the cGMP–PKG signaling pathway, the blood volume of SF and GF yaks may be different, which resulted in the different concentrations of precursors for fat deposition, including non-esterified fatty acid (NEFA), glucose (GLU), TG and cholesterol (CH). The metabolism of C16:0, C16:1, C17:0, C18:0, C18:1, C18:2 and C18:3 in yak subcutaneous fat was mainly realized under the regulation of the *INSIG1*, *ACACA*, *FASN*, *ELOVL6* and *SCD* genes, and TG synthesis was regulated by the *AGPAT2* and *DGAT2* genes. This study will provide a theoretical basis for yak genetic breeding and healthy feeding.

## 1. Introduction

Yak (*Bos grunniens*) is a kind of classic grazing livestock in Qinghai-Tibet Plateau and adjacent areas [1,2] that plays an essential role in the lives of local residents, providing resources such as animal-derived food, animal power and fuel [3]. After long-term natural selection, yaks have adapted to special living environments such as low temperatures and oxygen levels and possesses some unique physiological characteristics. Fat plays an important role in the life activities of yaks [4], such as survival under starvation, lactation and childbirth [5,6]. Yaks live through a cold season for more than six months of the year; grass is bad quality and scarce during this time. Moreover, the pregnancy and parturition of yaks mainly occurs in the cold season and female yaks suffer from long-term grass shortage and low temperature during pregnancy. Adequate fat deposition in female yaks can ensure a higher calving rate. After the calf is born, the fat deposition in female yaks is also one of the key factors for calf lactation. On the other hand, fat plays an important role in meat processing. If there is not enough fat in meat products, the overall meat quality will be reduced, leading to dryness and poor taste. Therefore, some subcutaneous or visceral fat is artificially added in order to develop tenderness and improve taste in the process of producing meat products.

The factors affecting the fat deposition in livestock are very complex and include genetics [7], diet [8,9], the feeding system [10,11] and gender [12,13]. In recent years, overgrazing has led to serious rangeland degradation on the Qinghai-Tibet Plateau and the survival and stocking rate of yaks has been seriously reduced [14]. Therefore, the nutrition management of yaks, especially the change in the traditional graze feeding (GF) method, is particularly important to the development of yak production. Stall feeding (SF) can significantly improve the growth performance and economic benefit of yaks; however, there are few reports on the effect of the feeding system on fat deposition in yaks.

Fat deposition in livestock is a dynamic equilibrium process that is regulated by fat synthesis, transport and decomposition. From the perspective of molecular biology, fat deposition is regulated by the expression of specific genes in time and space. Transcriptome analysis based on high-throughput sequencing technology possesses the advantages of higher resolution and sensitivity [15] and is being widely applied to studying the regulation of fat metabolism in beef cattle [16,17], dairy cows [18], chickens [19] and pigs [20]. Metabolites are the end product of gene expression, and the main metabolites in adipose tissue belong to lipids. Lipidomics can systematically analyze the changes in lipid composition in organisms [21] and has been successfully applied to exploring the fat characterization in cattle [22,23], dairy cattle [24], goats [25] and pigs [26]. The differential lipids in yak shanks and flanks have also been revealed by lipidomics approaches [27]. Plenty of lipid molecules with biological activity in specific substrates were found, and some new lipid functions in terms of livestock growth and development were clarified. The combination of lipidomics and transcriptomics can comprehensively and systematically reveal the physiological process of the fat metabolism in cattle [28,29], and RNA-Seq and lipidomics was used to reveal the different adipogenic processes between bovine perirenal and intramuscular adipocytes [28]. The regulatory mechanism of the feeding system to fat deposition in yak can also be revealed by the change in the lipidome and transcriptome in yak adipose tissue.

In this study, the deposition quantity of the subcutaneous fat in yaks under GF and SF was measured. The biochemical markers related to fat metabolism in yak serum under two feeding systems were detected. Furthermore, the transcriptomes and lipidomes in the subcutaneous fat of GF and SF yaks were detected by RNA sequencing (RNA-Seq) and non-targeted lipidomics based on ultrahigh-phase liquid chromatography tandem mass spectroscopy (UHPLC-MS), respectively. The differentially expressed genes (DEGs) and differential lipids (DLs) in the subcutaneous fat of SF and GF yaks were screened and annotated by gene ontology (GO) and Kyoto encyclopedia of genes and genome (KEGG) analysis. The correlation analysis of crucial DEGs and DLs was also carried out. Then, the regulatory mechanism of the feeding system on subcutaneous fat deposition in yaks was clarified from the angle of molecular biology. This study can provide a theoretical basis for yak genetics and breeding and efficient feeding.

## 2. Results

### 2.1. Thickness of Subcutaneous Fat in Yaks under Graze (GF) and Stall Feeding (SF)

The thickness of the subcutaneous fat in the backs of SF yaks was significantly greater than the value in the backs of GF yaks (7.28 ± 0.55 vs. 5.22 ± 0.78 mm, *p* < 0.01). Additionally, the thickness of the subcutaneous fat in the waist of SF yaks was significantly greater than the value in the waist of GF yaks (9.43 ± 1.13 vs. 6.08 ± 0.98 mm, *p* < 0.01).

### 2.2. Level of Biochemical Markers in the Sera of GF and SF Yaks 

The concentrations of glucose (GLU) (4.53 ± 0.28 vs. 4.06 ± 0.23 mmol/L), triglyceride (TG) (0.25 ± 0.05 vs. 0.18 ± 0.02 mmol/L), total cholesterol (TCH) (2.75 ± 0.14 vs. 2.14 ± 0.35 mmol/L), high density lipoprotein (HDL) (1.57 ± 0.20 vs. 1.24 ± 0.18 mmol/L), low density lipoprotein (LDL) (0.61 ± 0.09 vs. 0.44 ± 0.04 mmol/L), non-esterified fatty acid (NEFA) (0.22 ± 0.03 vs. 0.17 ± 0.01 mmol/L) and insulin (INS) (12.30 ± 1.45 vs. 10.02 ± 0.93 μIU/mL) in the sera of SF yaks were all higher than the respective values in the sera of GF yaks (*p* < 0.05).

### 2.3. Untargeted Metabolome Analyses for Subcutaneous Fat in GF and SF Yaks

**Figure 1:**
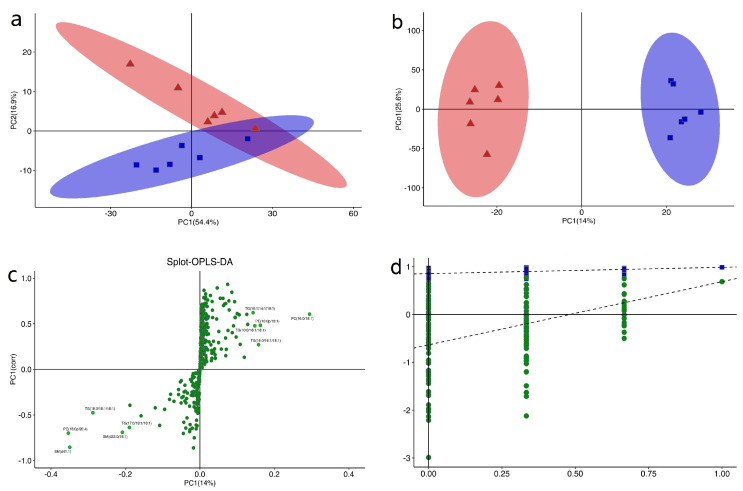
(**a**) The score plots of principal component analysis (PCA) of lipids in the subcutaneous fat of yaks under stall (SF) and graze feeding (GF). Red and blue represented the SF and GF groups, respectively. (**b**) The score plots of orthogonal partial least squares discriminant analysis (OPLS-DA) of lipids in the subcutaneous fat of yaks under SF and GF. (**c**) The splot diagram of OPLS-DA. (**d**) Permutation test of OPLS-DA model.

The score plot of principal component analysis (PCA) and orthogonal partial least squares discriminant analysis (OPLS-DA) for lipids in the subcutaneous fat of GF and SF yaks are shown in Figure 1a,b, respectively. The splot diagram of OPLS-DA is shown in Figure 1c. In order to prevent the overfitting of model, seven-fold cross validation and 200 response permutation testing were used to investigate the quality of the OPLS-DA model (Figure 1d). The values of R^2^Y and Q^2^Y were (0.0, 0.852) and (0.0, −0.636), respectively, and it can be found that the value of Q^2^Y was less than 0. Therefore, the OPLS-DA model was effective, stable and reliable. The lipids in the subcutaneous fat of SF and GF yaks were divided into two entirely different areas. It can be found there was a distinct difference in the lipids in the subcutaneous fat of SF and GF yaks, and the feeding system can lead to marked lipid perturbation in the subcutaneous fat of yaks.

**Figure 2:**
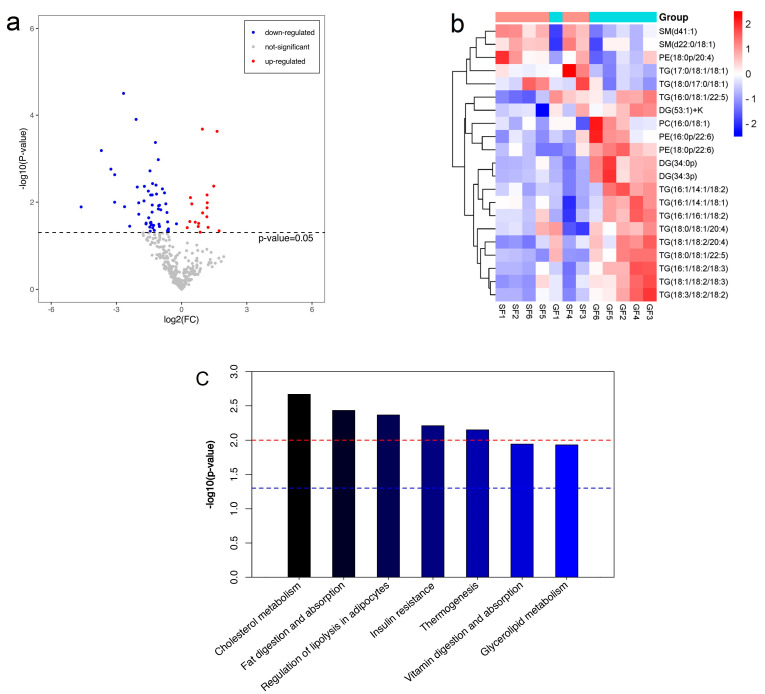
(**a**) The volcano plot of lipid abundance in the subcutaneous fat of SF yaks in contrast to GF yaks. The abscissa represents the value of log_2_ fold change (FC), and the ordinate represents the value of −log_10_ *P*. A point represents a gene. The points with FC > 2.0 and *p* < 0.05 or FC < 0.5 and *p* < 0.05 are shown in red or blue, respectively; non-different lipids (DLs) are shown in black. (**b**) The heat map of all DLs in the subcutaneous fat of SF and GF yaks. Each row represents a DL, and each column represents a sample. The color blocks at different positions represent the abundance of lipid molecules. Red represents high abundance in the subcutaneous fat of SF yaks, and blue represents low abundance in the subcutaneous fat of SF yaks. (**c**) The bar diagram of Kyoto encyclopedia of genes and genomes (KEGG) pathways for enrichment of DLs in the subcutaneous fat of SF and GF yaks.

A total of 408 lipid molecules in 13 classes were detected in the yak subcutaneous fat under the two feeding systems, including 14 ceramides (Cers), 1 cholesteryl ester (ChE), 52 diglycerides (DGs), 1 dimethylphosphatidylethanolamine (dMePE), 8 lysophosphatidylcholines (LPCs), 7 monoglycerides (MGs), 90 phosphatidylcholines (PCs), 32 phosphatidylethanolamines (PEs), 2 phosphatidylinositols (PIs), 2 phosphatidylserines (PSs), 27 sphingomyelins (SMs), 2 sphingoshines (SOs) and 171 triglycerides (TGs). The detailed information on lipid molecules is shown in Supplementary Table S1. The composition of lipid classes in the subcutaneous fat of SF and GF yaks is shown in Table 1. The predominant lipid classes in the subcutaneous fat of both SF and GF yaks were DGs, LPCs, PCs, PEs, SMs and TGs, whereas the abundance of DGs, PEs, SMs and TGs in yak subcutaneous fat was different between the SF and GF groups. Compared with the GF group, the percentage of PEs, SMs and TGs in the SF group was higher, whereas the percentage of DGs was lower. The volcano plot of lipid abundance in the subcutaneous fat of SF yaks in contrast to GF yaks is shown in Figure 2a. A total of 21 DLs were screened out, including 12 TGs, 3 PEs, 3 DGs, 2 SMs and 1 PC. The abundance of 15 DLs was downregulated in the subcutaneous fat of SF yaks, whereas 6 DLs were upregulated (Table 2). The heat map of DLs is shown in Figure 2b. Furthermore, TG(16:1/16:1/18:2), TG(18:0/18:1/20:4) and TG(18:1/18:2/20:4) were enriched in 7 KEGG pathways (Figure 2c), and the KEGG pathway involved in fat deposition included cholesterol metabolism (bom04979), fat digestion and absorption (bom04975), regulation of lipolysis in adipocytes (bom04923), insulin resistance (bom04931) and glycerolipid metabolism (bom00561).

### 2.4. Transcriptome Analysis for Subcutaneous Fat in GF and SF Yaks

**Figure 3:**
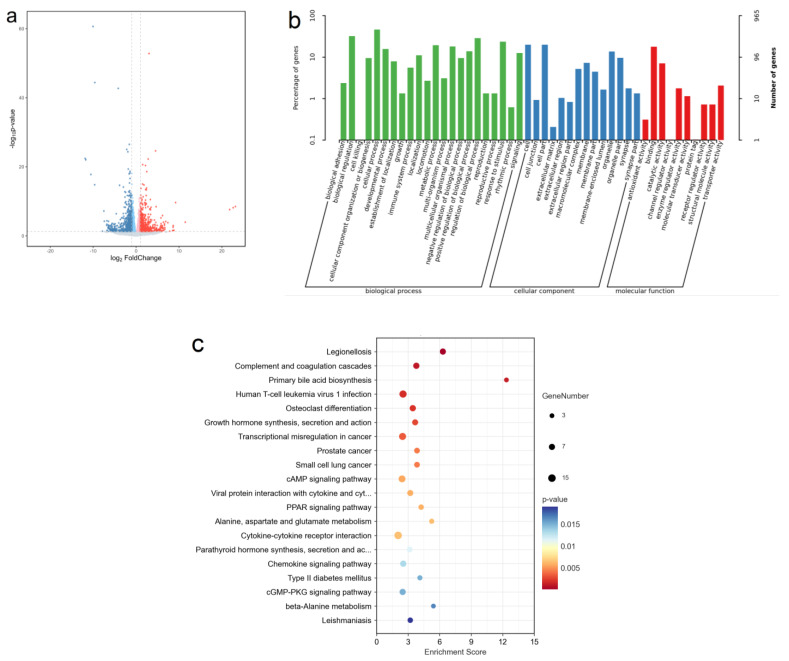
(**a**) Volcano plot of gene expression in the subcutaneous fat of SF yaks in contrast to GF yaks. The abscissa represents the value of log_2_ FC, and the ordinate represents the value of −log_10_ *P*. A point represents a gene. The points with FC > 2.0 and *p* < 0.05 or FC < 0.5 and *p* < 0.05 are shown in red or blue, respectively; non-differentially expressed genes (DEGs) are shown in black. (**b**) The top 30 terms for gene ontology (GO) enrichment for DEGs in the subcutaneous fat of SF yaks in contrast to GF yaks. (**c**) The top 20 terms for KEGG pathway enrichment analysis for DEGs in the subcutaneous fat of SF yaks in contrast to GF yaks.

A total of 17,269 genes were detected in the subcutaneous fat of yaks under the two feeding systems. A volcano plot of gene expression in the subcutaneous fat of SF yaks in contrast to GF yaks is shown in Figure 3a. A total of 677 DEGs were screened out, and their information is shown in Supplementary Table S2. Of them, the expression of 383 DEGs in the subcutaneous fat of SF yaks was downregulated, whereas the expression of 294 DEGs was upregulated. The DEGs involved in the regulation of fat metabolism included the *ELOVL6*, *GPD1*, *FASN*, *PCK1*, *INSIG1*, *GK2*, *ACACA*, *ME1*, *HK3*, *MOGAT1*, *LIPA*, *PGK2*, *SCD* and *ACOX2* genes, and their information is shown in Table 3. Because of the importance of the *DGAT2* (FC = 1.89) and *AGPAT2* (FC = 1.92) genes in regulating TG synthesis, these two genes were also discussed in this study. After GO enrichment, the biological processes for DEGs were focused on biological adhesion, biological regulation, growth and metabolic process (Figure 3b). Furthermore, these DEGs were enriched in 31 KEGG pathways (Supplementary Table S3), and the top 20 KEGG pathways are shown in Figure 3c. The KEGG pathways involved in the fat metabolism of adipose tissue included growth hormone synthesis, secretion and action (ko04935), the cAMP signaling pathway (ko04024), the PPAR signaling pathway (ko03320), cytokine–cytokine receptor interaction (ko04060), the cGMP–PKG signaling pathway (ko04022), insulin secretion (ko04911) and the adipocytokine signaling pathway (ko04920).

### 2.5. Quantitative Real-Time PCR (qPCR) Validation of Sequencing Data

The comparison results of quantitative real-time PCR (qPCR) and mRNA-Seq for the *ELOVL6*, *FASN*, *SCD* and *ACACA* genes in the subcutaneous fat of SF and GF yaks are shown in Supplementary Figure S1. The expressions of the *ELOVL6*, *FASN*, *SCD* and *ACACA* genes in the subcutaneous fat of SF yaks were upregulated compared with in GF yaks. All four DEGs in the subcutaneous fat of SF and GF yaks possessed similar expression patterns for qPCR and mRNA-Seq data, which indicated the reliability of the mRNA-Seq data for the subcutaneous fat of yaks under different feeding systems in this study.

### 2.6. Results of Correlation Analysis for Transcriptome and Lipidomics

**Figure 4:**
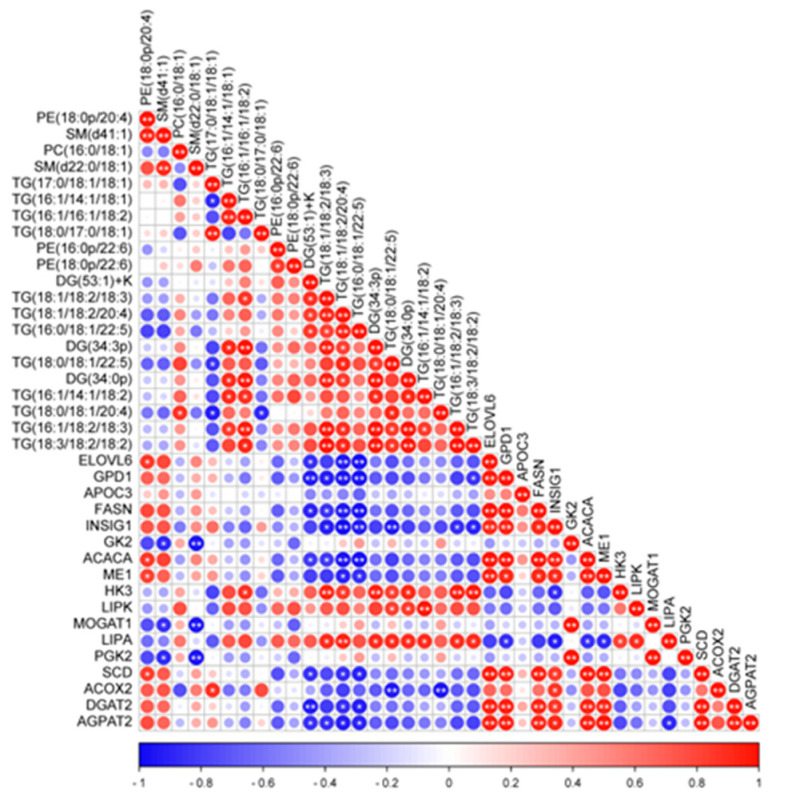
The correlation of crucial DLs and DEGs involved in regulating the fat deposition in yak subcutaneous fat. * represents *p* < 0.05 and ** represents *p* < 0.01. Circles with no * or ** represent *p* > 0.05. Red or blue represent positive or negative correlation, respectively. Deeper colors represent stronger correlations.

The results of Pearson analysis for crucial DEGs and DLs in the regulation of fat deposition in yak subcutaneous fat are shown in Figure 4. The expression of the *ELOVL6*, *GPD1*, *FASN*, *INSIG1, ACACA*, *SCD*, *DGAT2* and *AGPAT2* genes was negatively correlated with the abundance of DG(53:1)+K, TG(18:1/18:2/18:3), TG(18:1/18:2/20:4) and TG(16:0/-18:1/22:5); the expression of the *ELOVL6*, *ACACA*, *ME1* and *SCD* genes was positively correlated with the abundance of PE(18:0p/20:4); the expression of the *HK3* gene was positively correlated with the abundance of TG(16:1/16:1/18:2), TG(18:1/18:2/18:3), TG(18:1/18:2/20:4), DG(34:3p), DG(34:0p), TG(18:0/18:1/22:5), TG(16:1/18:2/18:3) and TG(18:3/18:2/18:2); the expression of the *LIPK* gene was positively correlated with the abundance of TG(18:0/18:1/22:5), DG(34:0p) and TG(16:1/14:1/18:2); the expression of the *MOGAT1* and *PGK2* genes was negatively correlated with the abundance of SM(d41:1) and SM(d22:0/18:1); the expression of the *ME1* gene was negatively correlated with the abundance of TG(18:1/18:2/20:4) and TG(16:0/18:1/22:5); the expression of the *INSIG1* gene was positively correlated with the abundance of TG(18:1/18:2/18:3), TG(18:1/18:2/20:4), TG(16:0/18:1/22:5) and TG(18:0/18:1/22:5).

## 3. Discussion

Changing the feeding system can lead to many effects in yaks from the macroscopic angle, such as dry matter intake, protein and energy levels, feeding behavior, digestive absorption and so on. Therefore, it was very difficult to explore the effect of the feeding system on fat deposition in yak subcutaneous fat from one of the above macro factors. However, all the above macro factors finally caused the changes in gene expression involved in the fat deposition in yaks, so it was feasible, reliable and accurate to explore the effect of the feeding system on the fat deposition in yak subcutaneous fat from the molecular level.

It was found that the capacity for fat deposition in SF yaks was stronger than that in GF yaks from the angle of subcutaneous fat thickness. Hormone [30] and adipocytokine [31,32] secretion play a crucial role in the regulation of fat deposition in cattle. In this study, many KEGG pathways for DEG enrichment were involved in hormone and adipocytokine secretion, such as insulin secretion (ko04911), the adipocytokine signaling pathway (04920), parathyroid hormone synthesis, secretion and action (ko04928), growth hormone synthesis and secretion and action (ko04935). Therefore, it was inferred the fat deposition in yaks under different feeding systems was also regulated by the hormone and adipocytokines from a macroscopic perspective. On the other hand, the KEGG pathways for DL enrichment involved in fat metabolism focused on cholesterol metabolism (bom04979), glycerolipid metabolism (bom00561), regulation of lipolysis in adipocytes (bom04923) and insulin resistance (bom04931). Therefore, the differences in fat deposition in the subcutaneous fat of yaks under different feeding systems was mainly caused by GLU, glycerolipid and CH metabolism under the regulation of hormone and adipocytokines. GLU, TG, TCH, HDL, LDL and NEFA are all the raw materials for lipid synthesis, and their levels in the sera of SF yaks were higher than the values in the sera of GF yaks. Apolipoprotein C3 mainly regulates the content of triglyceride-rich lipoproteins (TRLs) [33]. The *APOC3* gene positively acts in the transport of CH, TG and NEFA and is involved in the regulation of bovine subcutaneous fat deposition [34]. It can be inferred that the subcutaneous fatty tissue of SF yaks can obtain more GLU, CH, TG and NEFA in comparison with that inGF yaks, and the final result was that the capacity of fat deposition in SF yaks was stronger than that in GF yaks. The glycerolipid is the main ingredient of lipids in cattle [35], and the majority of DLs (except for two SMs) in yak subcutaneous fat belonged to the glycerolipid class. INS is related to blood glucose transport and utilization. The concentration of INS is increased with fattening phases and is positively correlated with the cattle marbling in late fattening phases [36]. The level of INS in the sera of SF yaks was higher than the value in the sera of GF yaks. CH has a role in fatty acid and lipid transport and is the precursor for hormone synthesis in cattle [37]. The primacy of glycerolipid metabolism and the functions of insulin and cholesterol in the regulation of yak fat deposition were also proved in this study.

Studies show that the cGMP–PKG signaling pathway (ko04022) mediates the regulation of relaxation and contraction of vascular smooth muscle cells [38,39]. The relaxation and contraction of the vascular smooth muscle cells in yaks under different feeding systems may be different, which led to different blood volumes and then the quantity of GLU, TG and NEFA arriving at the adipocytes in yak subcutaneous fat was different. The GLU from diet can be stored in adipose tissue in the form of TG by the glycolytic pathway and citric acid cycle [40]. The *ME1* gene acts in the release of NADPH and acetyl-CoA that are used in fatty acid biosynthesis [41]. The *INSIG1* gene is associated with fat metabolism and adipocyte differentiation in bovines [42], and INS can induce the expression of the *INSIG1* gene by regulating fatty acid synthesis [43]. The expression of the *ME1* gene was significantly correlated with the abundance of PE(18:0p/20:4), TG(18:1/18:2/20:4) and TG(16:0/18:1/22:5) and the expression of the *INSIG1* gene was positively correlated with the abundance of TG(18:1/18:2/18:3), TG(18:1/18:2/20:4), TG(16:0/18:1/22:5) and TG(18:0/18:1/22:5). Therefore, the fat deposition in yak subcutaneous fat due to the citric acid cycle was mainly realized under the regulation of the *INSIG1* and *ME1* genes.

The lipids in the subcutaneous fat of SF and GF yaks were mainly composed of LPCs, PCs, Pes and TGs, and the DLs were mainly formed by these fatty acids including C16:0, C16:1, C17:0, C18:0, C18:1, C18:2, C18:3, C20:4 and C22:5. Previous reports show that C16:0, C16:1, C18:1, C18:2, C18:3 are the major components of yak fat [44]. The level of fat deposition in cattle depends on the balance between lipid synthesis and decomposition, and the *ACACA*, *SCD*, *FASN* and *ACOX1* genes were screened out as the candidate genes for subcutaneous fat deposition in beef cattle [45]. The *ACACA* and *FASN* genes play an important role in the de novo synthesis of fatty acids in bovines [46]. The *ACACA* gene regulates the conversion of acetyl-CoA to malonyl-CoA; *FASN* regulates the conversion of malonyl-CoA to C16:0, then C16:0 translates into other fatty acids by elongation and desaturation reactions. The expression of the *FASN* and *ACACA* genes was significant correlation with the abundance of DG(53:1)+K, TG(18:1/18:2/18:3), TG(18:1/1-8:2/20:4) and TG(16:0/18:1/22:5) in yak subcutaneous fat. Therefore, the de novo synthesis of fatty acids in yak subcutaneous fat was mainly regulated by *ACACA* and *FASN*. *SCD* regulates the desaturation of saturated fatty acids (SFAs) in cattle [47], such as C16:0 to C16:1 and C18:0 to C18:1. The main function of the *ELOVL6* gene is to regulate the synthesis of monounsaturated fatty acids (MUFAs). Overexpression of the *ELOVL6* gene decreases the proportions of C14:0 and C16:0, whereas it increases the proportions of C18:0 and C20:4n6 in bovines [48]. The expression of *ELOVL6* and *SCD* was significantly correlated with the abundance of DG(53:1)+K, TG(18:1/18:2/18:3), TG(18:1/18:2/20:4), TG(16:0/18:1/22:5) and PE(18:0p/20:4) in yak subcutaneous fat. Therefore, the *ACACA*, *FASN*, *ELOVL6* and *SCD* genes were crucial in the regulation of fatty acid synthesis in yak subcutaneous fat.

The lipid composition in the adipose tissue of cattle is very complicated; the major component is TGs and lipidomic analyses showed that TGs comprised at least 50% of total lipids in bovine milk fat globule membranes [49]. In this study, it was found that TGs were also the major lipid component of yak subcutaneous fat. TG synthesis can be realized through two main pathways including the monoacylglycerol and glycerol 3 phosphate pathways [50]. *MOGAT1* regulates DG and TG synthesis and plays a key role in the route of TG synthesis in cattle [51]. The acylglycerophosphate acyltransferase (AGPAT) family plays a bridging role in TG synthesis [52], and the *AGPAT2* gene regulates the conversion of lysophosphatidic acid to phosphatidic acid. Diacylgycerol acyltransferases (DGATs) are the only rate-limiting enzyme in TG synthesis, and the *DGAT2* gene acts in the saturated fat deposition in the adipose tissue of Nellore cattle [53]. The thickness of the subcutaneous fat in SF yaks was significantly higher than the value for GF yaks, and the expression of the *DGAT2* and *AGPAT2* genes was significantly correlated with the abundance of DG(53:1)+K, TG(18:1/18:2/18:3), TG(18:1/18:2/20:4) and TG(16:0/18:1/22:5) in yak subcutaneous fat. Therefore, the *AGPAT2* and *DGAT2* genes were the key controlling genes for TG synthesis in yak subcutaneous fat.

## 4. Materials and Methods

### 4.1. Animals and Sample Collection

Twelve two-year-old healthy male yaks with the same genetic background were selected in Qinghai province, China, and divided in GF and SF groups. Each group contained six yaks. The yaks in GF group were only grazed on natural pasture with no supplements; the yaks in SF group were fed with total mixed ration (TMR) in the colony house. The nutritional aspects of the grass and TMR are shown in Supplementary Table S4. The GF yaks were grazed from 07:00 to 19:00 every day and could eat and drink freely during the grazing period. The SF yaks were fed twice a day and could also freely eat and drink. After being fed for six months, all twelve yaks were fasted for 24 h and cut off from water for 8 h. Twenty mL of blood was collected from the jugular vein of yaks in the morning of the slaughter day and centrifugated at 3000 r/min for 15 min at 4 °C using a KL05R refrigerated centrifuge (Kaida Inc., Changsha, China). Then, the serum samples were obtained and stored at −20 °C for analyzing the biochemistry index. Next, all yaks were humanely harvested at a commercial abattoir (Xiahua Meat Food Co., Ltd., Xining, China). The subcutaneous fat samples (12–13th rib level) were collected and placed in enzyme-free cryopreservation tubes and stored in liquid nitrogen.

### 4.2. Measure of Subcutaneous Fat Thickness in Yaks

The thickness of subcutaneous fat in yaks was measured with a Vernier caliper (Hengliang Inc., Shanghai, China) within 10 min after slaughter. In this study, the yak subcutaneous fat at two sites was chosen and measured, including the thickness of subcutaneous fat on the back (on both sides of midline of the dorsal at the 5–6th thoracic vertebra) and the thickness of subcutaneous fat on the waist (on both sides of midline at the cruciate region). The measure for the thickness of subcutaneous fat on each side of sites was carried out three times, and the average value was obtained.

### 4.3. Determination of Biochemical Markers in Yak Serum

The concentrations of GLU, TCH, TG, HDL, LDL and NEFA in yak serum was determined by the colorimetric method with a Mindray BS–420 automatic biochemical analyzer. The concentration of INS in yak serum was determined by enzyme-linked immunosorbent assay (ELISA). 

### 4.4. Lipid Extraction, Mass Spectrometry (Ms) Data and Bioinformatics

An amount of 20 mg of subcutaneous fatty tissue, 20 μL internal standard (the solution of Lyso PC17:0 in methanol, 0.1 mg/mL) and 300 μL of a solution of methanol–water (*v*:*v*, 1:1) were added into a tube, followed by adding two small steel balls. The tube was precooled at –20 °C for 2 min, then the sample was grinded at 60 Hz for 2 min using a Tissuelyser–48 grinding miller (Jingxing limited company, Shanghai, China). A total of 300 μL chloroform was transferred into the tube, then the fat sample was vortexed for 30 s with a TYXH–I vortex oscillator (hannuo company, shanghai, China) and extracted for 10 min with a F-060SD ultrasonic cleaner (Fuyang Technology Group Co. LTD, Shengzhen, China). The mixture was allowed to stand for 20 min at −20 °C and the residual mixture was treated once again according to the above steps. Another 200 μL of lower chloroform was combined with the previous solution, and a total of 400 μL chloroform solution was obtained. One hundred and fifty μL chloroform solution was transferred into a vial and dried, then the residue was redissolved in 500 μL of a solution of isopropanol and methanol (*v:v,* 1:1). The mixture was vortexed for 30 s and extracted with ultrasound for 3 min. The solution was transferred into a 1.5 mL centrifuge tube and allowed to for 2 h at −20 °C, followed by centrifugation at 12,000 r/min for 10 min. One hundred and fifty μL of supernatant was transferred into a vial for LC–MS analysis. Quality control (QC) samples were prepared by mixing the extract of all samples in equal volume.

The prepared samples were separated with an AB ExionLC HPLC coupled with an ACQUITY UPLC HSS T3 column (100 mm × 2.1 mm, 1.8 um) (Waters, Milford, CT, USA). The column temperature, flow rate, and sample size were 45 °C, 0.35 mL/min, and 2 μL, respectively. Mobile phase A was the solution of acetonitrile and water (*v:v*, 6:4) (containing 10 mmol/L ammonium formate); mobile phase B was the solution of isopropyl alcohol and acetonitrile (*v:v*, 9:1) (containing 10 mmol/L ammonium formate). The elution gradient was as follows: mobile phase B was held at 30% in 0.0–3.0 min, risen to 62% in 3.0–5.0 min, risen to 82% in 5.0–15.0 min, risen to 99% in 15.0–16.5 min, held at 99% in 16.5–18.0 min, reduced to 30% in 18.0–18.1 min, and held at 30% in 18.1–22.0 min. MS data was collected with an Q Exactive Plus mass spectrometer (Thermo Scientific™, Waltham, MA, USA) equipped with a heated electrospray ionization (ESI) source in positive and negative ion modes. The MS conditions were as follows: heater temp 300 °C, sheath gas flow rate 45 arb, aux gas flow rate 15 arb, sweep gas flow rate 1 arb, spray voltage 3.5 KV, capillary temp 320 °C, S-Lens RF level 50% and MS1 scan ranges 120–1800. The raw MS data was processed by software Lipid Search for MSn and the exact mass–to–charge ratio (*m/z*) of parent ions. The molecular structures of lipids in positive and negative mode were identified according to the parent ions and multi-stage mass spectrometry data of each individual sample. The results were aligned according to a certain retention time range and combined into a single report to sort out the original data matrix. In each sample, all peak signals were normalized. The extracted data was further processed by removing any peaks with a missing value (ion intensity = 0) in more than 50% of groups and by replacing the zero value by half of the minimum value. A data matrix was combined from the positive and negative ion data. The matrix was imported into R to carry out PCA in order to observe the overall distribution among the samples and the stability of the whole analysis process. OPLS–DA was utilized to distinguish the metabolites that differed between groups. To prevent overfitting, 7–fold cross-validation and 200 response permutation testing were used to evaluate the quality of the model. The variable importance of projection (VIP) values obtained from the OPLS–DA model were used to rank the overall contribution of each variable to the group discrimination. A two-tailed Student’s *t*-test was further used to verify whether the lipid differences between the SF and GF groups was significant. DLs were selected with VIP > 1.0 and *p* < 0.05.

### 4.5. RNA Extraction, Sequencing and Bioinformatics

The total RNA in yak subcutaneous fat was extracted using mirVana™ miRNA ISOlation Kit. Six hundred μL lysis/binding buffer was added into the subcutaneous fat sample and the mixture was homogenized, followed by adding 30 μL miRNA homogenate additive and standing in an ice bath for 10 min. The solution of acid phenol and chloroform (*v*:*v*, 1:1) was added into the tube and the mixture centrifuged at room temperature for 5 min. The supernatant was transferred out and 1.25 times the volume of 100% ethanol was added. The mixture was added into the column at room temperature and centrifuged at 13,000 r/min for 30 s; the supernatant was then discarded. Three hundred and fifty μL miRNA wash solution 1 was added into the column and the column centrifuged at 13,000 r/min for 30 s; the supernatant was then discarded. The solution of 10 μL DNase I and 70 μL buffer RDD QIAGEN was added on the column membrane, and the mixture was placed at room temperature for 15 min. Three hundred and fifty μL miRNA wash solution 1 was added to the centrifugal column, and the solution was centrifuged at 13,000 r/min for 30 s. The supernatant was discarded. The centrifugal column was put in the collection tube once again. Five hundred μL wash solution 2/3 was added in the column twice, and the solution was centrifuged at 13,000 r/min for 30 s. The supernatant was discarded. The centrifugal column was put in the collection tube once again. The empty column was centrifuged for 1 min. The centrifugal column was put into the collection tube and 100 μL of elution solution preheated at 95 °C was added to the center of the column. The column was allowed to stand for 2 min and then centrifuged at room temperature for 30 s. The liquid in the collection tube was the extracted total RNA. RNA integrity was evaluated using the Agilent 2100 Bioanalyzer (Agilent Technologies, Santa Clara, CA, USA). The samples with an RNA integrity number > 7 were subjected to the subsequent analysis. The libraries were constructed using TruSeq Stranded Total RNA with Ribo–Zero Gold. Then, these libraries were sequenced on the HiSeqTM 2500 sequencing platform (Illumina Inc., San Diego, CA, USA) and 150 bp paired-end reads were generated.

The raw reads were filtered with SortMeRNA and Trimmomatic software. The low-quality bases and N–bases or low-quality reads were removed. The quality of the processed data was detected with the software fastp, then the clean reads were obtained. Clean reads were mapped to the reference genome of yaks with hisat2 software. The read numbers in RNA-Seq analysis were normalized against reads per kilo base of transcripts per million (RPKM) to calculate the level of gene expression. The FPKM value of each gene was calculated using cufflinks, and the read counts of each gene were obtained by htseq-count. The counts were normalized using the estimateSizeFactors function of the DESeq (2012) R package. DEGs were screened using the nbinomTest function by calculated *p* and fold change (FC) for difference comparison. *p* < 0.05 and FC > 2 or <0.5 were set as the threshold for DEGs. GO and KEGG enrichment for DEGs was carried out using the hypergeometric distribution test.

### 4.6. Determination of qPCR for Candidate Genes Regulating Fat Deposition in Yaks

The qPCR method was used to detect the relative expression levels of four DEGs (*ELOVL6*, *FASN*, *SCD* and *ACACA*). The reference gene for qPCR was the *β-actin* gene. Quantification was performed with a two-step reaction process: reverse transcription (RT) and PCR. The RT reaction volume was 10 µL, which contained total RNA (0.5 μg), 5× TransScript All-in-one SuperMix for qPCR (5 μL) and gDNA Remover (0.5 μL). The reactions were carried out on a GeneAmp^®^ PCR System 9700 thermal cycler (Applied Biosystems, Foster City, CA, USA) for 15 min at 42 °C and 5 s at 85 °C. Real–time PCR was performed using a LightCycler^®^ 480 Real–Time PCR Instrument (Roche, Basel, Switzerland) with a 10 µL PCR mixture that included 0.2 µL cDNA, 5 µL 2 × PerfectStartTM Green qPCR SuperMix, 0.2 µL (10 µmol/L) primer and 3.6 µL nuclease-free water. Reactions were incubated in a 384-well optical plate (Roche, Basel, Switzerland) at 94 °C for 30 s, followed by 45 cycles of 94 °C for 5 s and 60 °C for 30 s. Each sample was run in triplicate for analysis. At the end of the PCR cycles, melting curve analysis was performed to validate the specific generation of the expected PCR product. Relative gene expression levels were determined using the 2^−∆∆Ct^ method.

### 4.7. Statistical Analysis

The thickness of subcutaneous fat in yaks and biochemical markers in yak serum were analyzed using one-way ANOVA and Student’s *t*-test in SPSS Statistics 22 (IBM, Chicago, IL, USA) software, and *p* < 0.05 was considered statistically significant. Correlations between crucial DLs and DEGs were carried on by Pearson correlation analysis also using SPSS 16.0. Significant differences were considered as *p* < 0.05, and a correlation coefficient > 0.8 was considered as a high correlation.

## 5. Conclusions

The capacity for fat deposition in the subcutaneous fat of yaks under SF was stronger than the yaks under GF, and there were also differences in the abundances of 12 TGs, 3 PEs, 3 DGs, 2 SMs and 1 PC in the subcutaneous fat between SF and GF yaks. These differences were mainly caused by cholesterol and glycerolipid metabolism and insulin action in yak adipose tissue. The blood volume of yaks under the two feeding systems may be different because of the mediation of the cGMP–PKG signaling pathway, which resulted in the concentration differences of the precursors for fat deposition in yaks, including NEFA, GLU and TG. The *INSIG1*, *ACACA*, *FASN*, *ELOVL6* and *SCD* genes play a crucial role in regulating fatty acid synthesis in the subcutaneous fat of yaks, and these fatty acids mainly include C16:0, C16:1, C17:0, C18:0, C18:1, C18:2, C18:3, C20:4 and C22:5. Furthermore, the TG synthesis in the subcutaneous adipose tissue of yaks was mainly realized under the regulation of the *AGPAT2* and *DGAT2* genes.

## Figures and Tables

**Table 1:** The composition of lipid classes in the subcutaneous fat of yaks under stall (SF) and graze feeding (GF).

Lipid Class	SF Group (Mean ± SD, %)	GF Group (Mean ± SD, %)	*p* Value
Cers	0.027 ± 0.015	0.029 ± 0.013	0.768
ChEs	0.002 ± 0.001	0.001 ± 0.0005	0.257
DGs	6.382 ± 2.292	13.548 ± 2.134	0.000
dMePEs	0.387 ± 0.178	0.406 ± 0.230	0.889
LPCs	13.368 ± 1.515	13.061 ± 3.038	0.844
MGs	0.067 ± 0.047	0.036 ± 0.005	0.176
PCs	20.205 ± 0.654	20.192 ± 1.542	0.986
PEs	10.953 ± 0.506	9.560 ± 0.879	0.012
PIs	0.379 ± 0.121	0.381 ± 0.125	0.975
PSs	0.0009 ± 0.0006	0.0012 ± 0.0007	0.514
SMs	8.506 ± 1.291	6.470 ± 1.219	0.028
SOs	0.007 ± 0.004	0.007 ± 0.003	0.984
TGs	39.72 ± 1.599	36.307 ± 1.536	0.006

SF: stall feeding; GF: graze feeding. SD: standard deviation. Cers: ceramides, ChEs: cholesteryl esters; DGs: diglycerides, dMePEs: dimethylphosphatidylethanolamines, LPCs: lysophosphatid-ylcholines, MGs: monoglycerides, PCs: phosphatidylcholines, PEs: phosphatidylethanolamines, PIs: phosphatidylinositols, PSs: phosphatidylserines, SMs: sphingomyelins, SOs: sphingoshines; TGs: triglycerides.

**Table 2:** The information for different lipids (DLs) in the subcutaneous fat of yaks under different feeding systems.

Lipid Molecule	Formula	VIP	*p* Value	FC
PE(18:0p/20:4)	C_43_H_76_O_7_N_1_P_1_	7.12	1.10 × 10^−2^	1.39
SM(d41:1)	C_46_H_93_O_6_N_2_P_1_	7.04	2.09 × 10^−4^	1.94
PC(16:0/18:1)	C_42_H_82_O_8_N_1_P_1_	5.93	3.15 × 10^−2^	0.85
SM(d22:0/18:1)	C_45_H_91_O_6_N_2_P_1_	4.20	7.85 × 10^−2^	1.33
TG(17:0/18:1/18:1)	C_56_H_104_O_6_N	3.82	2.79 × 10^−2^	1.30
TG(16:1/14:1/18:1)	C_51_H_92_O_6_N	2.87	4.12 × 10^−2^	0.66
TG(16:1/16:1/18:2)	C_53_H_94_O_6_N	2.54	4.65 × 10^−2^	0.66
TG(18:0/17:0/18:1)	C_56_H_106_O_6_N	2.18	3.86 × 10^−2^	1.20
PE(16:0p/22:6)	C_43_H_72_O_7_NP	2.13	3.17 × 10^−2^	0.32
PE(18:0p/22:6)	C_45_H_76_O_7_NP	2.02	3.91 × 10^−5^	0.40
DG(53:1)+K	C_56_H_108_O_5_K	2.02	4.53 × 10^−2^	0.64
TG(18:1/18:2/18:3)	C_57_H_98_ O_6_N	1.88	6.11 × 10^−5^	0.58
TG(18:1/18:2/20:4)	C_59_H_100_O_6_N	1.72	4.24 × 10^−4^	0.43
TG(16:0/18:1/22:5)	C_59_H_102_O_6_N	1.58	1.73 × 10^−2^	0.63
DG(34:3p)	C_37_H_66_ O_4_	1.57	4.33 × 10^−3^	0.31
TG(18:0/18:1/22:5)	C_61_H_106_O_6_N	1.50	3.15 × 10^−5^	0.16
DG(34:0p)	C_37_H_72_O_4_ Na	1.40	6.85 × 10^−3^	0.37
TG(16:1/14:1/18:2)	C_51_H_90_O_6_N	1.33	1.16 × 10^−2^	0.39
TG(18:0/18:1/20:4)	C_59_H_104_O_6_N	1.28	1.09 × 10^−2^	0.60
TG(16:1/18:2/18:3)	C_55_H_94_O_6_N	1.25	4.05 × 10^−3^	0.44
TG(18:3/18:2/18:2)	C_57_H_96_O_6_N	1.10	3.74 × 10^−5^	0.40

FC: fold change. SM: sphingomyelin; VIP: variable importance of projection; PC: phosphatidylcholine; TG: triglyceride; PE: phosphatidylethanolamine; DG: diglyceride.

**Table 3:** The information of crucial differentially expressed genes (DEGs) in the regulation of fat metabolism in yak subcutaneous fat.

Symbol	Description	FC	*p* Value
*ELOVL6*	ELOVL fatty acid elongase 6	5.56	1.40 × 10^−14^
*GPD1*	Glycerol-3-phosphate dehydrogenase 1	2.08	2.27 × 10^−4^
*APOC3*	Apolipoprotein c3	3.66 × 10^6^	2.45 × 10^−8^
*FASN*	Fatty acid synthase	10.00	6.51 × 10^−5^
*PCK1*	Phosphoenolpyruvate carboxykinase 1	3.57	1.78 × 10^−15^
*INSIG1*	Insulin induced gene 1	2.04	8.63 × 10^−7^
*GK2*	Glycerol kinase 2	0.01	9.85 × 10^−4^
*ACACA*	Acetyl-CoA carboxylase alpha	7.14	5.69 × 10^−23^
*ME1*	Malic enzyme 1	2.13	1.69 × 10^−5^
*INS*	Insulin	2.86	9.70 × 10^−10^
*HK3*	Hexokinase 3	0.24	1.23 × 10^−7^
*LIPK*	Lipase family member k	0.19	1.26 × 10^−10^
*MOGAT1*	Monoacylglycerol o-acyltransferase 1	0.02	3.13 × 10^−3^
*LIPA*	Lipase	0.34	1.22 × 10^−5^
*SLC2A4*	Solute carrier family 2 member 4	2.08	1.19 × 10^−3^
*PGK2*	Phosphoglycerate kinase 2	0.03	5.78 × 10^−4^
*SCD*	Stearoyl-CoA desaturase	6.67	3.72 × 10^−6^
*ACOX2*	Acyl-CoA oxidase 2	2.22	9.60 × 10^−9^
*DGAT2*	Diacylglycerol O-acyltransferase 2	1.89	3.23 × 10^−9^
*AGPAT2*	1-Acylglycerol-3-phosphate O-acyltransferase 2	1.92	1.59 × 10^−4^

## Data Availability

The original contributions presented in the study are included in the article/Supplementary Material, further inquiries can be directed to the corresponding authors.

## Supplementary Materials

The following supporting information can be downloaded at: https://www.mdpi.com/article/10.3390/ijms24087381/s1.
