# Supplementary material for: The Effect of the Feeding System on Fat Deposition in Yak Subcutaneous Fat
Source: Int J Mol Sci. 2023 Apr 17;24(8):7381. doi: 10.3390/ijms24087381 (PMC10138426; doi:10.3390/ijms24087381)
Supplement: Supplementary file 1 [file ijms-24-07381-s001.zip › Supplementary Table S4. The composition of the total mixed ration (TMR) and the content of common nutrition and major fatty aci.pdf]

Supplementary Table S4**.** The composition of the total mixed ration (TMR) and the content of common nutrition and major fatty acid in natural grass and TMR (air-dry basis).

**Item**	**TMR**	**Natural Grass**
Ingredient (%)		
Corn	19.20	-
Wheat bran	9.20	-
Whole corn silage	32.00	-
Oat Hay	28.00	-
Rapeseed meal	8.10	-
$NaHCO_3$	1.00	-
NaCl	1.50	-
Premix	1.00	-
Total	100.00	-
Common nutrition (%)		
Crude fat	4.52	2.63
Crude protein	16.96	11.93
Neutral detergent fiber	23.24	76.14
Acid detergent fiber	13.84	10.09
Calcium	0.79	5.22
Phosphorus	0.37	0.07
Fatty acids (%)		
C16:0	0.68	0.30
C18:0	0.21	0.09
C18:1	0.18	0.07
C18:2n6	0.58	0.29
C18:3n3	1.42	0.68

The ingredient of premix was 3000 IU VA, 500 IU VD3, 5 IU VE, 0.1 mg Se, 30 mg Fe, 18 mg Mn, 18 mg Zn and 3 mg Cu in per kg of the diets.
