# Supplementary figures and images for: The Effect of the Feeding System on Fat Deposition in Yak Subcutaneous Fat
Source: Int J Mol Sci. 2023 Apr 17;24(8):7381. doi: 10.3390/ijms24087381 (PMC10138426; doi:10.3390/ijms24087381)

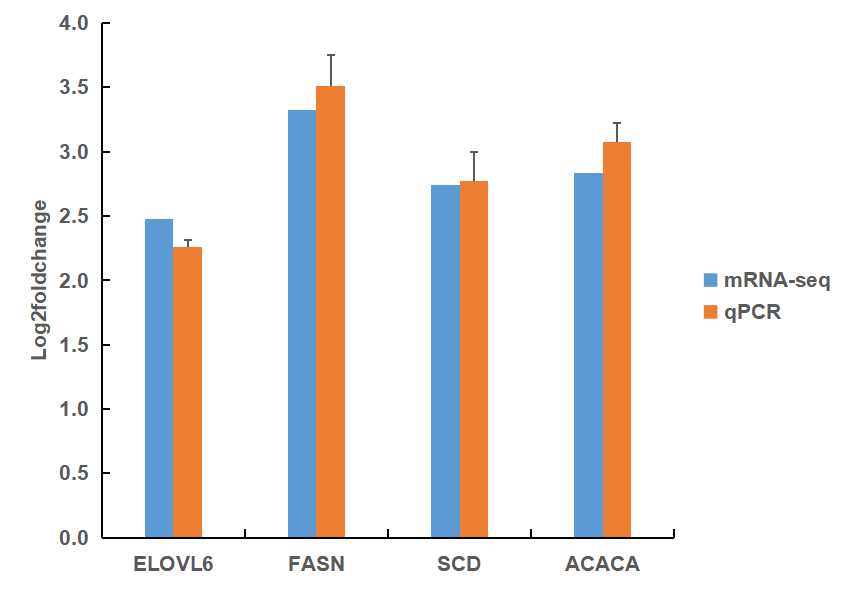

Supplement: Supplementary file 1 [file ijms-24-07381-s001.zip › Supplementary Figure S1 The comparison results of quantitative real-time PCR (qPCR) and mRNA-Seq for the ELOVL6, FASN, SCD and ACACA genes in the subcutaneous fat of SF and GF yaks.jpg]
